# Supplementary material for: SARS-CoV-2 Transmission in the Military during the Early Phase of the Pandemic—A Systematic Analysis
Source: Int J Environ Res Public Health. 2022 Jun 16;19(12):7418. doi: 10.3390/ijerph19127418 (PMC9224230; doi:10.3390/ijerph19127418)
Supplement: Supplementary file 1 [file ijerph-19-07418-s001.zip › ijerph-1734150-supplementary.pdf]

Supplementary Data for SARS-CoV-2 Exposure and Transmission in the Military during the Early Phase of the Pandemic: A Systematic Review

2.1 Summary of Findings Tables

*Rows highlighted in *italics* have potential overlapping cases, hence, are not included in most qualitative synthesis and meta-analyses

Table S1: Demographics of cases in included studies

Study	Male (%)	Age*	Race	Role	Unit	Other
Pirnay, J. P. (2020) [1]	9/9 (100%)	28 (23-45)[e]	-	Trainer (5/9, 55.6%)<br>Joint Support Detachment (2/9, 22.2%)<br>Medic (1/9, 11.1%)<br>Force Protection (1/9, 11.1%)	-	-
Escalera-Antezana, J. P. (2020) [2]	-	-	-	Sergeants & commissioned officers (1163/1261, 92.2%)<br>Soldiers & seamen (98/1261, 7.8%)	-	-
Pasqualotto, A. C. (2021) [3]	-	-	-	-	Military Police (52/52, 100%)	-
Halladay, J. (2020) [4]	-	-	-	-	-	-
Elhakim, M. (2020) [5]	1/1 (100%)	-	-	-	-	-
Paleiron, N. (2021) [6]	1112/1279 (86.9%)	28 [23-36]	-	-	Navy (1279/1279, 100%)	Smoking status<br>Never smoker (391/1279, 31%)<br>Former smoker (309/1279, 24%)<br>Current smoker (579/1279, 45%)<br>Current e-cigarette use (172/1279, 13%)
Chassery, L. (2021) [7]	-	-	-	-	*Navy (1148/1148, 100%)*	-
Joshi, R. K. (2020) [8]	-	-	-	-	-	-
Sasongko, S. (2021) [9]	-	-	-	-	Military/Police (144/144, 100%)	-
Nitecki, M. (2021) [10]	817/1338 (61.1%)	21 [20-23]	-	-	-	-
Segal, D. (2020) [11]	-	-	-	-	-	-
Talmy, T. (2021) [12]	84/119 (70.6%)	21 [19-25]	-	-	-	-
Segal, D. (2021) [13]	-	-	-	*26 Frontline combat units & essential military domains (12642/18719, 67.54%)<br>44 Rear administration &*	-	-

				support military units (6077/18719, 32.46%)		
Tsur, A. (2021) [14]	-	-	-	-	-	-
Di Nunno, D. (2020) [15]	75/81 (92.6%)	45.1 (10.4)	-	-	Army, Air force, Navy	-
Borud, E. K. (2021) [16]	-	-	-	New conscript (1/1, 100%)	-	-
Velasco, J. M. (2020) [17]	-	18-20 (3/515, 0.56%)<br>21-30 (164/515, 31.8%)<br>31-40 (179/515, 34.8%)<br>41-50 (136/515, 26.4%)<br>51-60 (33/515, 6.4%)	-	-	-	-
Oh, H. S. (2020) [18]	54/58 (93.1%)	27[b]	-	Enlisted soldiers (18/58, 31%)<br>Noncommisioned officers (18/58, 31%)<br>Officers (14/58, 24.1%)<br>Civilian employees (8/58, 13.8%)	Army (40/58, 67.2%)<br>Navy (3/58, 5.2%)<br>Air Force (15/58, 25.9%)	-
Wijesekara, N. (2021) [19]	-	-	-	-	Navy (1/1, 100%)	-
Baettig, S. J. (2020) [20]	2/2 (100%)	-	-	Recruit (2/2, 100%)	-	-
Bielecki, M. (2021) [21]	-	-	-	-	-	-
Crameri, G. A. G. (2020) [22]	*126/145 (86.9%)*	*Covid-19 Convalescents: 20.3 [19.8-21.5]*<br>*Asymptomatic: 20.8 [19.9-21.9]*	-	*Recruits (145/145, 100%)*	-	-
Handrick, S. (2020) [23]	1/1 (100%)	31	-	-	-	-
Stachow, E. (2021) [24]	-	-	-	-	-	-
Taylor, H. (2021) [25]	-	-	-	-	Army (7/7, 100%)	-
Clifton, G. T. (2021) [26]	4/6 (66.7%)	22 [20.3-23.0]	-	Registered nurse (1/6, 16.7%)<br>Medical support (1/6, 16.7%)[c]<br>Physician/physician assistant (0/6, 0%)<br>Other (4/6, 66.7%)	Army, 9th Hospital Center, 531st Hospital Center, 44th Medical Brigade	-
Elliott, B. P. (2021) [27]	-	-	-	-	-	-
Kasper, M. R. (2020) [28]	1042/1331 (78.3%)	27.1 (18-53)[a]	American Indian (34/1331, 2.6%)<br>Asian or Pacific Islander (106/1331, 8.0%)<br>Black (261/1331, 19.6%)<br>Hispanic (273/1331, 20.5%)<br>White (568/1331, 42.7%)<br>Other (88/1331, 6.6%)	Enlisted (1231/1331, 92.5%)<br>Officer (98/1331, 7.4%)<br>Unknown (2/1331, 0.2%)<br><br>Ship crew (786/1331, 59.1%)<br>Augmented crew (501/1331, 37.6%)<br>Unknown (44/1331, 3.3%)<br><br>Work department:<br>Air (65/1331, 4.9%)<br>Combat support division	Navy (1331/1331, 100%)	Tobacco/Nicotine user: (382/1331, 28.7%)

				(38/1331, 2.9%, 0.88 (0.58–1.33))<br>Deck (4/1331, 0.3%, 0.18 (0.07–0.52))<br>Engineering (67/1331, 5.0%, 1.85 (1.29–2.67))<br>Medical (8/1331, 0.6%, 0.76 (0.34–1.67))<br>Reactor (138/1331, 10.4%, 1.73 (1.29–2.36))<br>Supply (139/1331, 10.4%, 2.41 (1.78–3.26))<br>Weapons (94/1331, 7.1%, 2.70 (1.92–3.80))		
Kim, S. Y. (2021) [29]	-	-	-	-	-	-
Kline, J. D. (2020) [30]	1/1 (100%)	36	-	-	National Guard (1/1, 100%)	-
Kwon, P. O. (2020) [31]	1/1 (100%)	34	White (1/1, 100%)	Officer (1/1, 100%)	Army (1/1, 100%)	-
Lalani, T. (2021) [32]	5/13 (38.5%)	18-29 (8/13, 61.5%)<br>30-39 (2/13, 15.4%)<br>40+ (3/13, 23.1%)	White (6/13, 46.2%)<br>Black (2/13, 15.4%)<br>Hispanic (1/13, 7.7%)<br>Other (4/13, 30.8%)	Non-clinical (1/13, 7.7%)[d]<br>Corpsman (5/13, 38.5%)<br>Nurse (6/13, 46.2%)<br>Physician/medical assistant (1/13, 7.7%)	Navy + Other military healthcare workers	-
Letizia, A. G.(2020) [33]	-	-	-	Recruits (31/31, 100%)	Marine Corps (31/31, 100%)	-
Marcus, J. E. (2020) [34]	4/4 (100%)	-	-	Trainees (4/4, 100%)	Air Force (4/4, 100%)	-
Marcus, J. E. (2021) [35]	-	-	-	Trainees (269/269, 100%)	Air Force (269/269, 100%)	-
Servies, T. (2020) [36]	66/79 (83.5%)	20-29 (19/79, 24.1%)<br>30-39 (24/79, 30.4%)<br>40-49 (27/79, 34.2%)<br>50-59 (9/79, 11.4%)	White (53/79, 67.1%)<br>Black (10/79, 12.7%)<br>Other (6/79, 7.6%)<br>Unknown (10/79, 12.7%)	Junior enlisted (11/79, 13.9%)<br>Senior enlisted (25/79, 31.6%)<br>Junior officer (12/79, 15.2%)<br>Senior officer (25/79, 31.6%)<br>Warrant officer (1/79, 1.3%)<br>Unknown (5/79, 6.3%)	-	-
Smith, L. (2020) [37]	1/1 (100%)	21	-	Survival equipmentman (1/1, 100%)	-	-
Stanila, V. (2020) [38]	204/225 (90.7%)	< 20 (2/225, 0.9%)<br>20-24 (37/225, 16.4%)<br>25-29 (55/225, 24.4%)<br>30-39 (95/225, 42.2%)<br>40+ (36/225, 16.0%)	-	Junior enlisted (57/225, 25.3%)<br>Senior enlisted (85/225, 37.8%)<br>Junior officer (34/225, 15.1%)<br>Senior officer (29/225, 12.9%)<br>Warrant officer (20/225, 8.9%)	Army (160/225, 71.1%)<br>Navy (6/225, 2.7%)<br>Air Force (48/225, 21.3%)<br>Marine Corps (11/225, 4.9%)	-
Ghoddusi, F. (2021) [39]	1/1 (100%)	28	African American (1/1, 100%)	-	Army (1/1, 100%)	-
Letizia, A. G. (2021) [40]	-	-	-	Recruits (1079/1079, 100%)	Marine Corps (1079/1079, 100%)	-
Sikorski, C. S. (2021) [41]	-	-	-	Enlisted (3/6, 50%)<br>Officer (3/6, 50%)	Navy (6/6, 100%)	-
Alvarado, G. R. (2020) [42]	*572/736 (77.7%)*	*25 [22-31]*	-	-	*Navy (736/736, 100%)*	-

Kebisek, J. (2020) [43]	*175/219 (79.9%)*	*<25 (34/219, 15.5%)<br>25-34 (76/219, 34.7%)<br>35-44 (67/219, 30.6%)<br>45+ (42/219, 19.2%)*	-	-	*Army (219/219, 100%)*	-
Payne, D. C. (2020) [44]	*190/238 (79.8%)*	*18–24 (77/238, 32.4%)<br>25–29 (50/238, 21.0%)<br>30–39 (87/238, 36.6%)<br>40–59 (24/238, 10.1%)*	*American Indian/Alaska Native or Native Hawaiian/Other Pacific Islander (9/238, 3.8%)<br>Asian (13/238, 5.5%)<br>Black (25/238, 10.5%)<br>Hispanic/Latino (47/238, 19.7%)<br>Other/Unknown (2/238, 0.8%)<br>White (142/238, 59.7%)*	-	*Navy (238/238, 100%)*	-
Stidham, R. A. (2020) [45]	*Active duty (23987/39703, 60.4%)<br>Recruits (5491/39703, 13.8%)<br>Reserve/Guard (1880/39703, 4.7%)<br>Cadets (326/39703, 0.8%)*	*Active duty/ Recruits/Reserve or Guard/Cadets<br>15-19 (2517/39703, 6.3%)/ (4004/39703, 10.1%)/ (332/39703, 0.8%)/ (221/39703, 0.6%)<br>20-24 (11033/39703, 27.8%)/ (2008/39703, 5.1%)/ (537/39703, 1.4%)/ (205/39703, 0.5%)<br>25-29 (6944/39703, 17.5%)/ (571/39703, 1.4%)/ (432/39703, 1.1%)/ (2/39703, 0.005%)<br>30-34 (3952/39703, 10.0%)/ (176/39703, 0.4%)/ (377/39703, 0.9%)/ 0<br>35-39 (2777/39703, 7.0%)/ (41/39703, 0.1%)/ (275/39703, 0.7%)/ 0<br>40-44 (1403/39703, 3.5%)/ (7/39703, 0.02%)/ (193/39703, 0.5%)/ 0<br>45-49 (760/39703, 1.9%)/ 0/ (152/39703, 0.4%)/ 0<br>50-54 (363/39703, 0.9%)/ 0/ (136/39703, 0.3%)/ 0<br>55-59 (147/39703, 0.4%)/ 0/ (55/39703, 0.1%)/ 0<br>60-64 (54/39703, 0.1%)/ 0/ (8/39703, 0.02%)/ 0<br>65+ (20/39703, 0.05%)/ 0/ (1/39703, 0.003%)/ 0*	*Active duty/ Recruits/Reserve or Guard<br>Non-Hispanic White (13585/39703, 34.2%)/ (3275/39703, 8.2%)/ (1150/39703, 2.9%)<br>Non-Hispanic Black (6651/39703, 16.8%)/ (1167/39703, 2.9%)/ (539/39703, 1.4%)<br>Hispanic (6269/39703, 15.8%)/ (1408/39703, 3.5%)/ (494/39703, 1.2%)<br>Asian/Pacific Islander (1039/39703, 2.6%)/ (235/39703, 0.6%)/ (33/39703, 0.08%)<br>Native American/Alaska Native (255/39703, 0.6%)/ (48/39703, 0.1%)/ (9/39703, 0.02%)<br>Other (1214/39703, 3.1%)/ (203/39703, 0.5%)/ (150/39703, 0.4%)<br>Unknown (957/39703, 2.4%)/ (471/39703, 1.2%)/ (123/39703, 0.3%)*	*Active duty/Reserve or Guard<br>Combat specific (4123/39703, 10.4%)/ (183/39703, 0.5%)<br>Motor transport (1049/39703, 2.6%) (76/39703, 0.2%)<br>Pilot/air crew (997/39703, 2.5%)/ (85/39703, 0.2%)<br>Repair/engineering (8503/39703, 21.4%) /(583/39703, 1.5%)<br>Communications/intelligence (6480/39703, 16.3%)/(572/39703, 1.4%)<br>Healthcare (2756/39703, 6.9%)/(203/39703, 0.5%)<br>Others/Unknown (6062/39703, 15.3%)/(796/39703, 2.0%)*	*Active duty/ Recruits/Reserve or Guard/Cadets<br>Army (12213/39703, 30.8%)/ (3823/39703, 9.6%)/ (1557/39703, 3.9%)/ (116/39703, 0.3%)<br>Navy (7742/39703, 19.5%)/ (1378/39703, 3.5%)/ (95/39703, 0.2%)/ (214/39703, 0.5%)<br>Air Force (6346/39703, 16.0%)/ (561/39703, 1.4%)/ (787/39703, 2.0%)/ (92/39703, 0.2%)<br>Marine Corps (3423/39703, 8.6%)/ (1039/39703, 2.6%)/ (50/39703, 0.1%)/ 0<br>Coast Guard/USPHS (246/39703, 0.6%)/ (6/39703, 0.02%)/ (9/39703, 0.02%)/ (6/39703, 0.02%)*	-
Vick, D. J. (2021) [46]	-	-	-	-	-	-

[*] Median [IQR], Mean (SD), unless indicated otherwise; actual age given for case report

[a] Mean (range), IQR: 22-31

[b] Median

[c] Medical support refers to licensed practical nurses, medics, therapists, and other specialists who typically interact with patients.

[d] Non-clinical refers to liaison officer, public affairs office, quarterdeck or radio operators, pharmacy staff, oxygen plant operators, flight deck and administrative staff in patient care areas

[e] Median (range)

Table S2: Clinical characteristics of cases in included studies

**Study**	**Total**	**Cough**	**Rhinorrhea**	**Nasal congestion**	**Sore throat**	**Cold like symptoms**	**Anosmia**	**Ageusia**	**Anosmia/ Ageusia**	**Headache**	**Body aches**	**Myalgia**	**Dyspnea**	**Chest pain**	**Chills**	**Weakness**	**Fatigue**	**Fever**	**Nausea**	**Vomiting**	**Diarrhea**	**Abdominal pain**	**Gastrointestinal symptoms**	**Cutaneous manifestation**
Pirnay, J. P. (2020)[c] [1]	9	-	-	-	-	-	-	-	4	2	-	2	-	-	-	-	2	3	-	-	1	-	-	-
Escalera-Antezana, J. P. (2020) [2]	1261	-	-	-	-	-	-	-	-	-	-	-	-	-	-	-	-	-	-	-	-	-	-	-
Pasqualotto, A. C. (2021) [3]	-	-	-	-	-	-	-	-	-	-	-	-	-	-	-	-	-	-	-	-	-	-	-	-
Halladay, J. (2020) [4]	55	-	-	-	-	-	-	-	-	-	-	-	-	-	-	-	-	-	-	-	-	-	-	-
Elhakim, M. (2020) [5]	-	-	-	-	-	-	-	-	-	-	-	-	-	-	-	-	-	-	-	-	-	-	-	-
Paleiron, N. (2021) [6]	1279	286	322	-	-	-	614	499	-	666	523	523	437	-	-	575	-	322	-	-	-	-	-	-
Chassery, L. (2021) [7]	*1148*	-	-	-	-	-	-	-	-	-	-	-	-	-	-	-	-	-	-	-	-	-	-	-
Joshi, R. K. (2020) [8]	27	-	-	-	-	-	-	-	-	-	-	-	-	-	-	-	-	-	-	-	-	-	-	-
Sasongko, S. (2021) [9]	144	-	-	-	-	121	-	-	-	-	-	-	-	-	-	-	-	-	-	-	-	-	52	-
Nitecki, M. (2021) [10]	1338	-	-	-	-	-	-	-	-	-	-	-	-	-	-	-	-	-	-	-	-	-	-	-
Segal, D. (2020) [11]	-	-	-	-	-	-	-	-	-	-	-	-	-	-	-	-	-	-	-	-	-	-	-	-
Talmy, T. (2021)[f] [12]	119	75	30	-	32	-	61	47	-	48	-	28	23	7	8	41	-	45	11	-	22	7	-	5
Segal, D. (2021) [13]	-	-	-	-	-	-	-	-	-	-	-	-	-	-	-	-	-	-	-	-	-	-	-	-
Tsur, A. (2021) [14]	*3*	-	-	-	-	-	-	-	-	-	-	-	-	-	-	-	-	-	-	-	-	-	-	-
Di Nunno, D. (2020) [15]	81	-	-	-	-	-	-	-	-	-	-	-	-	-	-	-	-	-	-	-	-	-	-	25
Borud, E. K. (2021) [16]	1	-	-	-	-	-	-	-	-	-	-	-	-	-	-	-	-	-	-	-	-	-	-	-
Velasco, J. M. (2020) [17]	-	-	-	-	-	-	-	-	-	-	-	-	-	-	-	-	-	-	-	-	-	-	-	-
Oh, H. S. (2020) [18]	58	-	-	-	-	-	-	-	-	-	-	-	-	-	-	-	-	-	-	-	-	-	-	-
Wijesekara, N. (2021) [19]	-	-	-	-	-	-	-	-	-	-	-	-	-	-	-	-	-	-	-	-	-	-	-	-
Baettig, S. J. (2020) [20]	2	2	-	-	-	-	-	-	-	1	-	-	-	-	-	-	-	1	-	-	-	-	-	-
Bielecki, M. (2021) [21]	255	-	-	-	-	-	-	-	-	-	-	-	-	-	-	-	-	-	-	-	-	-	-	-
Crameri, G. A. G. (2020) [22]	*145*	-	-	-	-	-	-	-	-	-	-	-	-	-	-	-	-	-	-	-	-	-	-	-
Handrick, S. (2020) [23]	1	-	-	-	-	-	-	-	-	-	-	-	-	-	-	-	-	-	-	-	-	-	-	-
Stachow, E. (2021)[e] [24]	21	15	3	2	2	-	-	-	13	2	-	2	7	-	-	-	4	7	-	-	-	-	-	-

Taylor, H. (2021) [25]	7	-	-	-	-	-	-	-	-	-	-	-	-	-	-	-	-	-	-	-	-	-	-	-
Clifton, G. T. (2021) [26]	6	2	3	-	3	-	2	-	-	-	-	-	1	-	-	-	-	2	-	-	1	-	-	-
Elliott, B. P. (2021) [27]	1	-	-	-	-	-	-	-	-	-	-	-	1	-	-	-	-	-	-	-	-	-	-	-
Kasper, M. R. (2020)[a] [28]	1331	452	-	332	-	-	-	-	321	516	-	-	154	-	-	-	-	100	-	-	-	-	-	-
Kim, S. Y. (2021) [29]	-	-	-	-	-	-	-	-	-	-	-	-	-	-	-	-	-	-	-	-	-	-	-	-
Kline, J. D. (2020) [30]	1	1	1	1	1	-	-	-	-	-	-	-	1	-	1	-	-	1	1	1	1	-	-	-
Kwon, P. O. (2020) [31]	1	1	-	-	-	-	1	1	-	-	-	1	1	-	-	-	1	-	1	1	1	-	-	-
Lalani, T. (2021) [32]	13	3	-	4	-	-	1	-	-	4	-	2	-	-	-	-	1	1	-	-	1	-	-	-
Letizia, A. G.(2020) [33]	-	-	-	-	-	-	-	-	-	-	-	-	-	-	-	-	-	-	-	-	-	-	-	-
Marcus, J. E. (2020) [34]	4	-	-	-	-	-	-	-	-	-	-	-	-	-	-	-	-	-	-	-	-	-	-	-
Marcus, J. E. (2021) [35]	269	-	-	-	-	-	-	-	-	-	-	-	-	-	-	-	-	-	-	-	-	-	-	-
Servies, T. (2020) [36]	68	-	-	-	-	-	-	-	-	-	-	-	-	-	-	-	-	-	-	-	-	-	-	-
Smith, L. (2020)[d] [37]	1	1	-	1	1	-	1	1	-	1	-	-	-	-	-	-	-	-	-	-	1	-	-	-
Stanila, V. (2020) [38]	56	24	-	15	10	-	7	4	-	14	-	6	6	-	-	-	22	10	-	-	4	-	-	-
Ghoddusi, F. (2021) [39]	1	1	-	-	-	-	1	-	-	-	-	-	0	-	1	-	-	0	1	1	1	-	-	-
Letizia, A. G. (2021) [40]	1079	-	-	-	-	-	-	-	-	-	-	-	-	-	-	-	-	-	-	-	-	-	-	-
Sikorski, C. S. (2021) [41]	-	-	-	-	-	-	-	-	-	-	-	-	-	-	-	-	-	-	-	-	-	-	-	-
Alvarado, G. R. (2020) [42]	*736*	*332*	-	-	*195*	*386*	*275*	*230*	-	*252*	*146*	-	*23*	*87*	*52*	-	*40*	*55*	-	-	-	-	*65*	-
Kebisek, J. (2020) [43]	*219*	-	-	-	-	-	-	-	-	-	-	-	-	-	-	-	-	-	-	-	-	-	-	-
Payne, D. C. (2020)[b] [44]	*238*	*86*	*108*	-	*81*	-	-	-	*119*	*129*	-	*109*	*46*	*40*	*85*	-	*107*	*89*	*40*	*11*	*47*	*39*	-	-
Stidham, R. A. (2020) [45]	*39703*	-	-	-	-	-	-	-	-	-	-	-	-	-	-	-	-	-	-	-	-	-	-	-
Vick, D. J. (2021) [46]	-	-	-	-	-	-	-	-	-	-	-	-	-	-	-	-	-	-	-	-	-	-	-	-

[a] Chest pain/pressure reported by 199 individuals
[b] Palpitation reported by 19 individuals
[c] Sore throat/nasal congestion reported by 2 individuals (unsure if its either symptom or both)
[d] Reduced exercise tolerance reported by case
[e] One report of arthralgia, back pain and chest tightness each
[f] Loss of appetite reported by 16 individuals

References

1. Pirnay JP, Selhorst P, Cochez C, Petrillo M, Claes V, Van der Beken Y, Verbeken G, Degueldre J, T'Sas F, Van den Eede G *et al*: **Study of a SARS-CoV-2 Outbreak in a Belgian Military Education and Training Center in Maradi, Niger**. *Viruses* 2020, **12**(9).
2. Escalera-Antezana JP, Mariaca-Cerball CA, Alvarado-Arnez LE, Balderrama-Saavedra MA, Bonilla-Aldana DK, Rodriguez-Morales AJ: **Incidence of SARS-CoV-2/COVID-19 in military personnel of Bolivia**. *BMJ Mil Health* 2020.
3. Pasqualotto AC, de Castro Pereira P, Dalla Lana DF, Schwarzbold AV, Ribeiro MS, Riche CVW, Castro CPP, Korsack PL, Ferreira PEB, de Domingues GC *et al*: **COVID-19 seroprevalence in military police force, Southern Brazil**. *PLoS ONE* 2021, **16**(4 April 2021).
4. Halladay J, Bélanger SAH: **Fast facts on COVID-19 and the Canadian Armed Forces**. *J Mil Veteran Fam Health* 2020, **6**(2):7-8.
5. Elhakim M, Tourab SB, Zouiten A: **COVID-19 pandemic in Djibouti: Epidemiology and the response strategy followed to contain the virus during the first two months, 17 March to 16 May 2020**. *PLoS One* 2020, **15**(12):e0243698.
6. Paleiron N, Mayet A, Marbac V, Perisse A, Barazzutti H, Brocq FX, Janvier F, Bertrand D, Bylicki O: **Impact of Tobacco Smoking on the risk of COVID-19.A large scale retrospective cohort study**. *Nicotine Tob Res* 2021.
7. Chassery L, Texier G, Pommier De Santi V, Chaudet H, Bonnardel N, Pellegrin L: **A COVID-19 outbreak on board ship: Analysis of the sociotechnical system of epidemiological management in the French Navy**. *Saf Sci* 2021, **140**:105296.
8. Joshi RK, Ray RK, Adhya S, Chauhan VPS, Pani S: **Spread of COVID-19 by asymptomatic cases: evidence from military quarantine facilities**. *BMJ Mil Health* 2020.
9. Sasongko S, Nurrokhmawati Y, Rahayu DF: **Characteristics of inpatients suspected covid-19 based on swab results rt-pcr in dustira army hospital (Period on August, 15th – November, 15th 2020)**. *Annals of the Romanian Society for Cell Biology* 2021, **25**(4):7710-7720.
10. Nitecki M, Taran B, Ketko I, Geva G, Yosef R, Toledo I, Twig G, Avramovitch E, Gordon B, Derazne E *et al*: **Self-reported symptoms in healthy young adults to predict potential coronavirus disease 2019**. *Clin Microbiol Infect* 2021, **27**(4):618-623.
11. Segal D, Rotschield J, Ankory R, Kutikov S, Moaddi B, Verhovsky G, Benov A, Twig G, Glassberg E, Fink N *et al*: **Measures to Limit COVID-19 Outbreak Effects Among Military Personnel: Preliminary Data**. *Mil Med* 2020, **185**(9-10):e1624-e1631.
12. Talmy T, Tsur A, Shabtay O: **Duration of SARS-CoV-2 detection in Israel Defense Forces soldiers with mild COVID-19**. *J Med Virol* 2021, **93**(2):608-610.
13. Segal D, Arzi YI, Bez M, Cohen M, Rotschield J, Fink N, Karp E: **Promoting Compliance to COVID-19 Vaccination in Military Units**. *Mil Med* 2021.
14. Tsur A, Furer A, Avramovich E, Karp E, Twig G, Bader T, Almakias M, Fink N: **SARS-CoV-2 Epidemic in the Israeli Defense Force-Lessons Learned From Our rt-PCR Screening Policy**. *Mil Med* 2021.
15. Di Nunno D, Laghi A, Troiani G, Marseglia B, Anelli L, Farina E, Tarricone F, Mammana G, Catalano C, Bonito A *et al*: **Cutaneous manifestations in an Italian military COVID hub**. *G Ital Dermatol Venereol* 2020, **155**(5):632-635.
16. Borud EK, Nakstad ER, Håberg SE, Lind A, Fadum EA, Taxt AM, Steens A, Gjein GE, Sunde MW, Iversen P *et al*: **Severe acute respiratory syndrome coronavirus 2 prevalence in 1170 asymptomatic Norwegian conscripts**. *Health Sci Rep* 2021, **4**(1):e233.
17. Velasco JM, Navarro FC, Diones PC, Villa V, Valderama MT, Tabinas H, Chua D, Rosa RD, Turao-Agoncillo MM, Timbol JC *et al*: **SARS-CoV-2 Among Military and Civilian Patients, Metro Manila, Philippines**. *Mil Med* 2020.
18. Oh HS, Woong S: **Strict Containment Strategy and Rigid Social Distancing Successfully Contained COVID-19 in the Military in South Korea**. *Mil Med* 2020, **185**(11-12):476-479.
19. Wijesekara N, Herath N, Kodituwakku K, Herath HDB, Ginige S, Ruwanpathirana T, Kariyawasam M, Samaraweera S, Herath A, Jayawardena S *et al*: **Predictive modelling for COVID-19 outbreak control: lessons from the navy cluster in Sri Lanka**. *Mil Med Res* 2021, **8**(1):31.
20. Baettig SJ, Parini A, Cardona I, Morand GB: **Case series of coronavirus (SARS-CoV-2) in a military recruit school: clinical, sanitary and logistical implications**. *BMJ Mil Health* 2020.

21. Bielecki M, Züst R, Siegrist D, Meyerhofer D, Crameri GAG, Stanga Z, Stettbacher A, Buehrer TW, Deuel JW: **Social Distancing Alters the Clinical Course of COVID-19 in Young Adults: A Comparative Cohort Study**. *Clin Infect Dis* 2021, **72**(4):598-603.
22. Crameri GAG, Bielecki M, Züst R, Buehrer TW, Stanga Z, Deuel JW: **Reduced maximal aerobic capacity after COVID-19 in young adult recruits, Switzerland, May 2020**. *Euro Surveill* 2020, **25**(36).
23. Handrick S, Bestehorn-Willmann M, Eckstein S, Walter MC, Antwerpen MH, Naija H, Stoecker K, Wölfel R, Ben Moussa M: **Whole genome sequencing and phylogenetic classification of Tunisian SARS-CoV-2 strains from patients of the Military Hospital in Tunis**. *Virus Genes* 2020, **56**(6):767-771.
24. Stachow E, Warden S, Cockcroft K, Schofield S: **Symptomatology of COVID-19, rhinovirus and undifferentiated viral infections in a static military population**. *BMJ Mil Health* 2021.
25. Taylor H, Wall W, Ross D, Janarthanan R, Wang L, Aiano F, Ellis J, Gopal R, Andrews N, Patel M *et al*: **Cross sectional investigation of a COVID-19 outbreak at a London Army barracks: Neutralising antibodies and virus isolation**. *Lancet Reg Health Eur* 2021, **2**:100015.
26. Clifton GT, Pati R, Krammer F, Laing ED, Broder CC, Mendu DR, Simons MP, Chen HW, Sugiharto VA, Kang AD *et al*: **SARS-CoV-2 Infection Risk Among Active Duty Military Members Deployed to a Field Hospital - New York City, April 2020**. *MMWR Morb Mortal Wkly Rep* 2021, **70**(9):308-311.
27. Elliott BP, Buchek GM, Koroscil MT: **Characteristics, Treatment, and Outcomes of Patients With Severe or Life-threatening COVID-19 at a Military Treatment Facility-A Descriptive Cohort Study**. *Mil Med* 2021.
28. Kasper MR, Geibe JR, Sears CL, Riegodedios AJ, Luse T, Von Thun AM, McGinnis MB, Olson N, Houskamp D, Fenequito R *et al*: **An Outbreak of Covid-19 on an Aircraft Carrier**. *N Engl J Med* 2020, **383**(25):2417-2426.
29. Kim SY, Lee K, Tussey JB, Dougherty EJ, Williams SC, Abrams RB, Murray CK: **Responding to COVID-19 Among U.S. Military Units in South Korea: The U.S. Forces Korea's Operation Kill the Virus**. *Mil Med* 2021.
30. Kline JD, Donovan AE: **Sentinel Case of COVID-19 at Fort Stewart, GA in a National Guard Soldier Participating in Annual Training: A Case Report**. *Mil Med* 2020, **185**(11-12):e2158-e2161.
31. Kwon PO, Fisher NA, Mancuso JD: **Sars-cov-2 and influenza coinfection in a deployed military setting—two case reports**. *Med Surveill Mon Report* 2020, **27**(12):18-23.
32. Lalani T, Lee TK, Laing ED, Ritter A, Cooper E, Lee M, Baker M, Baldino T, McAdoo T, Phogat S *et al*: **SARS-CoV-2 Infections and Serologic Responses Among Military Personnel Deployed on the USNS COMFORT to New York City During the COVID-19 Pandemic**. *Open Forum Infect Dis* 2021, **8**(2):ofaa654.
33. Letizia AG, Ramos I, Obla A, Goforth C, Weir DL, Ge Y, Bamman MM, Dutta J, Ellis E, Estrella L *et al*: **SARS-CoV-2 Transmission among Marine Recruits during Quarantine**. *N Engl J Med* 2020, **383**(25):2407-2416.
34. Marcus JE, Frankel DN, Pawlak MT, Casey TM, Blackwell RS, Tran FV, Dolan MJ, Yun HC: **COVID-19 Monitoring and Response Among U.S. Air Force Basic Military Trainees - Texas, March-April 2020**. *MMWR Morb Mortal Wkly Rep* 2020, **69**(22):685-688.
35. Marcus JE, Frankel DN, Pawlak MT, Casey TM, Cybulski RJ, Jr., Enriquez E, Okulicz JF, Yun HC: **Risk Factors Associated With COVID-19 Transmission Among US Air Force Trainees in a Congregant Setting**. *JAMA Netw Open* 2021, **4**(2):e210202.
36. Servies T: **Characteristics of U.S. Army Beneficiary Cases of COVID-19 in Europe, 12 March 2020-17 April 2020**. *Msmr* 2020, **27**(12):9-13.
37. Smith L, Hodges C, Pratt M, Porter IM: **Case Report: COVID-19 Patient With Chief Complaint of Anosmia and Ageusia; a Unique Perspective on Atypical Symptomatology and Management in the Military**. *Mil Med* 2020, **185**(11-12):e2176-e2179.
38. Stanila V, Wells N, Ziadeh C, Stahlman S: **Air Evacuation of Service Members for COVID-19 in U.S. Central Command and U.S. European Command From 11 March 2020 Through 30 September 2020**. *Msmr* 2020, **27**(12):14-17.
39. Ghoddusi F, Garcia D, Harroun T: **Post-COVID Multisystem Inflammatory Syndrome in the Deployed Environment**. *Mil Med* 2021.
40. Letizia AG, Ge Y, Vangeti S, Goforth C, Weir DL, Kuzmina NA, Balinsky CA, Chen HW, Ewing D, Soares-Schanoski A *et al*: **SARS-CoV-2 seropositivity and subsequent infection risk in healthy young adults: a prospective cohort study**. *Lancet Respir Med* 2021.
41. Sikorski CS, Scheel MD, Harris SM, Nefczyk JA: **COVID-19 Contact Tracing in an Overseas U.S. Military Population**. *Mil Med* 2021.

42. Alvarado GR, Pierson BC, Teemer ES, Gama HJ, Cole RD, Jang SS: **Symptom Characterization and Outcomes of Sailors in Isolation After a COVID-19 Outbreak on a US Aircraft Carrier**. *JAMA Netw Open* 2020, **3**(10):e2020981.
43. Kebisek J, Forrest LJ, Maule AL, Steelman RA, Ambrose JF: **Special report: Prevalence of selected underlying health conditions among active component Army service members with coronavirus disease 2019, 11 February-6 April 2020**. *Msmr* 2020, **27**(5):50-54.
44. Payne DC, Smith-Jeffcoat SE, Nowak G, Chukwuma U, Geibe JR, Hawkins RJ, Johnson JA, Thornburg NJ, Schiffer J, Weiner Z *et al*: **SARS-CoV-2 Infections and Serologic Responses from a Sample of U.S. Navy Service Members - USS Theodore Roosevelt, April 2020**. *MMWR Morb Mortal Wkly Rep* 2020, **69**(23):714-721.
45. Stidham RA, Stahlman S, Salzar TL: **Cases of Coronavirus Disease 2019 and Comorbidities Among Military Health System Beneficiaries, 1 January 2020 through 30 September 2020**. *Msmr* 2020, **27**(12):2-8.
46. Vick DJ: **Evaluation of glucose-6-phosphate dehydrogenase (G6PD) status in US military and VA patients with COVID-19 infection**. *BMJ Mil Health* 2021, **167**(2):144.

2.2 Tables from Meta-Analysis

Table S3. Incidence of COVID-19 in military populations (Figures used in original study analysis)

**Study**	**Incidence**	**LCI 95%**	**HCI 95%**	**weight (%)**
Baettig, S. J. (2020)	0.014285714	0.0002245	0.042583419	0.019773665
Bielecki, M. (2021)	0.436643836	0.396618399	0.477085622	0.082261263
Borud, E. K. (2021)	0.000897666	0	0.003852749	0.156852314
Clifton, G. T. (2021)	0.017857143	0.005955021	0.035295313	0.047358281
Escalera-Antezana, J. P. (2020)	0.02519984	0.023844571	0.026591558	7.04259149
Halladay, J. (2020)	0.032352941	0.02443559	0.041326748	0.239324684
Joshi, R. K. (2020)	0.794117647	0.639640095	0.915718102	0.004855455
Kasper, M. R. (2020)	0.278510149	0.265889105	0.291309209	0.672656469
Lalani, T. (2021)	0.030092593	0.015797794	0.048565615	0.060869112
Letizia, A. G.(2020)	0.018149883	0.012312951	0.02507041	0.240450586
Marcus, J. E. (2020)	0.000982077	0.00020723	0.00223411	0.573295559
Marcus, J. E. (2021)	0.025670388	0.022726689	0.028787961	1.474862112
Nitecki, M. (2021)	0.054921599	0.052095365	0.057818013	3.428725436
Oh, H. S. (2020)	9.68E-05	7.34E-05	0.000123456	84.30203183
Paleiron, N. (2021)	0.757701422	0.73696234	0.777854234	0.23763583
Pirnay, J. P. (2020)	0.128571429	0.058935474	0.218489347	0.009922017
Stachow, E., S. (2021)	0.265822785	0.173618049	0.369341939	0.011188658
Velasco, J. M. (2020)	0.102142007	0.093931545	0.110655561	0.709670519
Letizia, A. G. (2021)	0.378463697	0.360741418	0.396349703	0.401313928
Pasqualotto, A. C. (2021)	0.032663317	0.024457265	0.041996728	0.224124998
Sasongko, S. (2021)	0.832369942	0.772748143	0.884633139	0.024418014
Taylor, H. (2021)	0.027559055	0.010393581	0.051885176	0.035817778
Pooled	0.001931524	0	0.091827054	100
Statistics				
I-squared	99.93021483	99.92592786	99.93425368	
Cochran's Q	30092.35136			
Chi2, p	0			

Table S4. Incidence of COVID-19 in military populations (Largest figures mentioned in each study)

**Study**	**Incidence**	**LCI 95%**	**HCI 95%**	**weight (%)**
Baettig, S. J. (2020)	0.014285714	0.0002245	0.042583419	0.019513916
Bielecki, M. (2021)	0.436643836	0.396618399	0.477085622	0.081180668
Borud, E. K. (2021)	0.000897666	0	0.003852749	0.154791882

Clifton, G. T. (2021)	2.8049243065393E-318	0	0.002907108	0.082152892
Escalera-Antezana, J. P. (2020)	0.02519984	0.023844571	0.026591558	6.950079097
Halladay, J. (2020)	0.032352941	0.02443559	0.041326748	0.236180884
Joshi, R. K. (2020)	0.794117647	0.639640095	0.915718102	0.004791673
Kasper, M. R. (2020)	0.278510149	0.265889105	0.291309209	0.663820366
Lalani, T. (2021)	0.015	0.008809923	0.022742895	0.166736343
Letizia, A. G.(2020)	0.016954194	0.012848089	0.021612174	0.467014538
Marcus, J. E. (2020)	0.000982077	0.00020723	0.00223411	0.565764675
Marcus, J. E. (2021)	0.025713478	0.022785981	0.028812574	1.474654826
Nitecki, M. (2021)	0.047637478	0.045294278	0.050036724	4.306325425
Oh, H. S. (2020)	9.68E-05	7.34E-05	0.000123456	83.19462944
Paleiron, N. (2021)	0.757701422	0.73696234	0.777854234	0.234514215
Pirnay, J. P. (2020)	0.128571429	0.058935474	0.218489347	0.00979168
Stachow, E., S. (2021)	0.265822785	0.173618049	0.369341939	0.011041682
Velasco, J. M. (2020)	0.102142007	0.093931545	0.110655561	0.700348195
Letizia, A. G. (2021)	0.378463697	0.360741418	0.396349703	0.396042217
Pasqualotto, A. C. (2021)	0.032663317	0.024457265	0.041996728	0.221180863
Sasongko, S. (2021)	0.832369942	0.772748143	0.884633139	0.024097256
Taylor, H. (2021)	0.027559055	0.010393581	0.051885176	0.035347271
Pooled	0.002044531	0	0.085501551	100
Statistics				
I-squared	99.93083032	99.92659572	99.93482063	
Cochran's Q	30360.12195			
Chi2, p	0			

Table S5: COVID-19 Incidence by Populations with Possible Exposure Locally or Overseas

**Study or subgroup**	**Incidence**	**LCI 95%**	**HCI 95%**	**weight (%)**
Local				
Baettig, S. J. (2020)	0.014285714	0.0002245	0.04258342	0.172195089
Bielecki, M. (2021)	0.436643836	0.3966184	0.47708562	0.716356082
Borud, E. K. (2021)	0.000897666	0	0.00385275	1.365917628
Clifton, G. T. (2021)	0.017857143	0.00595502	0.03529531	0.412410302
Escalera-Antezana, J. P. (2020)	0.02519984	0.02384457	0.02659156	61.32902743
Halladay, J. (2020)	0.032352941	0.02443559	0.04132675	2.084112092
Lalani, T. (2021)	0.030092593	0.01579779	0.04856561	0.530066733
Letizia, A. G.(2020)	0.018149883	0.01231295	0.02507041	2.093916795

Marcus, J. E. (2020)	0.000982077	0.00020723	0.00223411	4.992431995
Marcus, J. E. (2021)	0.025670388	0.02272669	0.02878796	12.84354759
Letizia, A. G. (2021)	0.378463697	0.36074142	0.3963497	3.494763676
Pasqualotto, A. C. (2021)	0.032663317	0.02445726	0.04199673	1.951748607
Local subgroup	0.03027915	0	0.12529533	91.98649402
Overseas				
Kasper, M. R. (2020)	0.278510149	0.26588911	0.29130921	5.857696998
Paleiron, N. (2021)	0.757701422	0.73696234	0.77785423	2.069405038
Pirnay, J. P. (2020)	0.128571429	0.05893547	0.21848935	0.086403941
Overseas subgroup	0.398455459	0	0.95868076	8.013505978
Pooled	0.045779986	0	0.22032107	100
Statistics				
I-squared	99.87702962	99.8654299	99.8876295	
Cochran's Q	11384.85502			
Chi2, p	0			

Table S6: COVID-19 Incidence by Deployment

**Study or subgroup**	**Incidence**	**LCI 95%**	**HCI 95%**	**weight (%)**
Deployment				
Clifton, G. T. (2021)	0.017857143	0.005955021	0.035295313	1.065294816
Halladay, J. (2020)	0.032352941	0.02443559	0.041326748	5.383458647
Kasper, M. R. (2020)	0.278510149	0.265889105	0.291309209	15.13098536
Lalani, T. (2021)	0.030092593	0.015797794	0.048565615	1.369212505
Paleiron, N. (2021)	0.757701422	0.73696234	0.777854234	5.345468935
Pirnay, J. P. (2020)	0.128571429	0.058935474	0.218489347	0.223189553
Deployment subgroup	0.267750424	0	0.715085989	28.51760981
No Deployment				
Baettig, S. J. (2020)	0.014285714	0.0002245	0.042583419	0.444796201
Bielecki, M. (2021)	0.436643836	0.396618399	0.477085622	1.850415512
Borud, E. K. (2021)	0.000897666	0	0.003852749	3.52829442
Joshi, R. K. (2020)	0.794117647	0.639640095	0.915718102	0.109220419
Letizia, A. G.(2020)	0.018149883	0.012312951	0.02507041	5.408785121
Marcus, J. E. (2020)	0.000982077	0.00020723	0.00223411	12.89592402
Marcus, J. E. (2021)	0.025670388	0.022726689	0.028787961	33.17609814
Letizia, A. G. (2021)	0.378463697	0.360741418	0.396349703	9.027305105

Pasqualotto, A. C. (2021)	0.032663317	0.024457265	0.041996728	5.041551247
No Deployment subgroup	0.043673441	0	0.179270684	71.48239019
Pooled	0.090518017	0	0.262766819	100
Statistics				
I-squared	99.85765617	99.84345109	99.87057229	
Cochran's Q	9835.340093			
Chi2, p	0			

Table S7: COVID-19 Incidence by Local or Overseas Deployment

**Study or subgroup**	**Incidence**	**LCI 95%**	**HCI 95%**	**weight (%)**
Local				
Clifton, G. T. (2021)	0.017857143	0.005955021	0.035295313	3.735568384
Halladay, J. (2020)	0.032352941	0.02443559	0.041326748	18.8776643
Lalani, T. (2021)	0.030092593	0.015797794	0.048565615	4.801287744
Local subgroup	0.03030349	0.023653851	0.037420595	27.41452043
Overseas				
Kasper, M. R. (2020)	0.278510149	0.265889105	0.291309209	53.05839254
Paleiron, N. (2021)	0.757701422	0.73696234	0.777854234	18.74444938
Pirnay, J. P. (2020)	0.128571429	0.058935474	0.218489347	0.782637655
Overseas subgroup	0.398445431	0	0.958680756	72.58547957
Pooled	0.267731996	0	0.715085989	100
Statistics				
I-squared	99.84011451	99.81127074	99.86455004	
Cochran's Q	3127.238043			
Chi2, p	0			

Table S8: Incidence of Symptomatic Individuals among COVID-19 Cases

**Study**	**Incidence**	**LCI 95%**	**HCI 95%**	**weight (%)**
Bielecki, M. (2021)	0.419607843	0.359610478	0.48081367	4.420415225
Clifton, G. T. (2021)	0.666666667	0.238814873	0.984581891	0.112456747
Di Nunno, D. (2020)	1	0.97885118	1	1.410034602
Joshi, R. K. (2020)	0	0	0.062982805	0.475778547
Kasper, M. R. (2020)	0.570247934	0.543549032	0.596744175	23.03633218
Lalani, T. (2021)	0.384615385	0.135570546	0.66690378	0.233564014
Nitecki, M. (2021)	1	0.998715593	1	23.15743945

Oh, H. S. (2020)	1	0.970505847	1	1.012110727
Paleiron, N. (2021)	0.865519937	0.846255599	0.883686913	22.1366782
Pirnay, J. P. (2020)	0.555555556	0.219875443	0.868152098	0.164359862
Servies, T. (2020)	1	0.974824909	1	1.185121107
Stachow, E. (2021)	1	0.919292916	1	0.371972318
Stanila, V. (2020)	0.803571429	0.688024498	0.898455217	0.977508651
Letizia, A. G. (2021)	0.321594069	0.294034973	0.349787998	18.67647059
Sasongko, S. (2021)	1	0.988085825	1	2.5
Taylor, H. (2021)	1	0.766154117	1	0.129757785
Pooled	0.778982533	0.439114518	1	100
Statistics				
I-squared	99.51381118	99.43924937	99.57845867	
Cochran's Q	3085.221112			
Chi2, p	0			

Table S9: Incidence of Hospitalisation among COVID-19 Cases

**Study**	**Incidence**	**LCI 95%**	**HCI 95%**	**weight (%)**
Bielecki, M. (2021)	0.00392157	0	0.01677235	5.65328023
Di Nunno, D. (2020)	1	0.97885118	1	1.80329682
Kasper, M. R. (2020)	0.01728024	0.01090494	0.02504868	29.4612236
Marcus, J. E. (2021)	0	0	0.00638349	5.96304901
Paleiron, N. (2021)	0.08365911	0.06908221	0.09948596	28.3106538
Pirnay, J. P. (2020)	0	0	0.18415391	0.21020024
Servies, T. (2020)	0.04411765	0.00564683	0.1088565	1.51565439
Letizia, A. G. (2021)	0	0	0.00159264	23.8853856
Sasongko, S. (2021)	1	0.98808583	1	3.19725633
Pooled	0.04425557	0	0.25345845	100
Statistics				
I-squared	99.566674	99.4773294	99.6407461	
Cochran's Q	1846.18499			
Chi2, p	0			

Table S10: Incidence of Deaths among COVID-19 Cases

**Study**	**Incidence**	**LCI 95%**	**HCI 95%**	**weight (%)**
Bielecki, M. (2021)	0	0	0.006733582	4.629461859
Escalera-Antezana, J. P. (2020)	0.019032514	0.012150324	0.027379757	22.8574017

Halladay, J. (2020)	0	0	0.031094471	1.00561696
Kasper, M. R. (2020)	0.000751315	0	0.003225161	24.12574742
Oh, H. S. (2020)	0	0	0.029494153	1.059974633
Paleiron, N. (2021)	0	0	0.001343644	23.18354774
Pirnay, J. P. (2020)	0	0	0.184153913	0.172132633
Servies, T. (2020)	0	0	0.025175091	1.241166878
Talmy, T. (2021)	0	0	0.014411329	2.165247327
Letizia, A. G. (2021)	0	0	0.001592636	19.55970284
Pooled	0.002507072	0	0.008531565	100
Statistics				
I-squared	83.64382593	71.44243984	90.63209782	
Cochran's Q	55.02509306			
Chi2, p	1.20E-08			

Table S11: Incidence of Males among COVID-19 Cases

**Study**	**Incidence**	**LCI 95%**	**HCI 95%**	**weight (%)**
Clifton, G. T. (2021)	0.66666667	0.238815	0.984582	0.1469259
Di Nunno, D. (2020)	0.92592593	0.85708	0.974581	1.8422242
Kasper, M. R. (2020)	0.78287002	0.760303	0.804621	30.097197
Lalani, T. (2021)	0.38461538	0.135571	0.666904	0.3051537
Nitecki, M. (2021)	0.61061286	0.584324	0.636585	30.255425
Oh, H. S. (2020)	0.93103448	0.848683	0.984676	1.3223327
Paleiron, N. (2021)	0.86942924	0.850391	0.887359	28.92179
Pirnay, J. P. (2020)	1	0.815846	1	0.2147378
Servies, T. (2020)	0.83544304	0.744681	0.909985	1.7970163
Stanila, V., N. (2020)	0.90666667	0.864855	0.941549	5.0971971
Pooled	0.77568345	0.634954	0.902078	100
Statistics				
I-squared	97.2020127	96.1081	97.98846	
Cochran's Q	321.65979			
Chi2, p	0			

Table S12: Incidence of Ageusia among Symptomatic COVID-19 Cases

**Study**	**Incidence**	**LCI 95%**	**HCI 95%**	**weight (%)**
Paleiron, N. (2021)	0.450767841	0.421539213	0.480167226	87.03339882

Stanila, V. (2020)	0.088888889	0.020051418	0.192937111	3.575638507
Talmy, T. (2021)	0.394957983	0.308709084	0.484582469	9.390962672
Pooled	0.430710069	0.162071272	0.711075063	100
Statistics				
I-squared	93.60584847	84.69996096	97.32777324	
Cochran's Q	31.27858312			
Chi2, p	1.61E-07			

Table S13: Incidence of Anosmia among Symptomatic COVID-19 Cases

**Study**	**Incidence**	**LCI 95%**	**HCI 95%**	**weight (%)**
Paleiron, N. (2021)	0.55465221	0.52527672	0.58383815	86.65884194
Lalani, T. (2021)	0.2	0	0.67287378	0.430359937
Stanila, V. (2020)	0.15555556	0.06219373	0.27811194	3.560250391
Talmy, T. (2021)	0.51260504	0.42252051	0.60228451	9.350547731
Pooled	0.53426009	0.253292	0.80937097	100
Statistics				
I-squared	91.1285839	80.3962498	95.9853588	
Cochran's Q	33.8164727			
Chi2, p	2.17E-07			

Table S14: Incidence of Anosmia/Ageusia among Symptomatic COVID-19 Cases

**Study**	**Incidence**	**LCI 95%**	**HCI 95%**	**weight (%)**
Kasper, M. R. (2020)	0.422924901	0.3879677	0.4582719	96.567069
Pirnay, J. P. (2020)	0.8	0.3271262	1	0.6993007
Stachow, E. (2021)	0.619047619	0.3996553	0.8170974	2.73363
Pooled	0.430515628	0.1827024	0.6881361	100
Statistics				
I-squared	64.2909269	0	89.767666	
Cochran's Q	5.600817458			
Chi2, p	0.060785213			

*Anosmia and/or Ageusia was not defined by these studies

Table S15: Incidence of Cough among Symptomatic COVID-19 Cases

**Study**	**Incidence**	**LCI 95%**	**HCI 95%**	**weight (%)**
Kasper, M. R. (2020)	0.595520422	0.5603562	0.6302015	36.886838

Lalani, T. (2021)	0.6	0.1420489	0.977994	0.26712
Paleiron, N. (2021)	0.258355917	0.2329795	0.2845704	53.788247
Stachow, E. (2021)	0.714285714	0.4996437	0.8904025	1.0441962
Stanila, V. (2020)	0.533333333	0.3858808	0.6779721	2.2098106
Talmy, T. (2021)	0.630252101	0.541302	0.7150164	5.8037882
Pooled	0.41104983	0.1211129	0.7197717	100
Statistics				
I-squared	98.04497957	97.103392	98.68049	
Cochran's Q	255.7518035			
Chi2, p	0			

Table S16: Incidence of Diarrhea among Symptomatic COVID-19 Cases

**Study**	**Incidence**	**LCI 95%**	**HCI 95%**	**weight (%)**
Lalani, T. (2021)	0.2	0	0.6728738	3.125
Pirnay, J. P. (2020)	0.2	0	0.6728738	3.125
Stanila, V. (2020)	0.088888889	0.0200514	0.1929371	25.852273
Talmy, T. (2021)	0.18487395	0.119763	0.2601152	67.897727
Pooled	0.16329688	0.1119965	0.2219354	100
Statistics				
I-squared	0	0	82.968792	
Cochran's Q	2.697195076			
Chi2, p	0.440704179			

Table S17: Incidence of Dyspnea among Symptomatic COVID-19 Cases

**Study**	**Incidence**	**LCI 95%**	**HCI 95%**	**weight (%)**
Kasper, M. R. (2020)	0.202898551	0.175015	0.2322848	36.985634
Paleiron, N. (2021)	0.394760614	0.3661449	0.4237413	53.932311
Stachow, E. (2021)	0.333333333	0.145172	0.5514056	1.0469929
Stanila, V. (2020)	0.133333333	0.0471721	0.2506277	2.2157292
Talmy, T. (2021)	0.193277311	0.1268562	0.2695582	5.8193328
Pooled	0.301319491	0.1391691	0.4773353	100
Statistics				
I-squared	95.84358498	92.758282	97.614408	
Cochran's Q	96.23678043			
Chi2, p	0			

Table S18: Incidence of Fatigue among Symptomatic COVID-19 Cases

Study	Incidence	LCI 95%	HCI 95%	weight (%)
Lalani, T. (2021)	0.2	0	0.67287378	7.051282051
Pirnay, J. P. (2020)	0.4	0.02200597	0.8579511	7.051282051
Stachow, E. (2021)	0.19047619	0.04640867	0.39069928	27.56410256
Stanila, V. (2020)	0.48888889	0.3430346	0.63568106	58.33333333
Pooled	0.38113404	0.19126191	0.58376218	100
Statistics				
I-squared	49.9852356	0	83.4555063	
Cochran's Q	5.9982288			
Chi2, p	0.11169644			

Table S19: Incidence of Fever among Symptomatic COVID-19 Cases

Study	Incidence	LCI 95%	HCI 95%	weight (%)
Kasper, M. R. (2020)	0.131752306	0.108581004	0.156786055	36.78856866
Lalani, T. (2021)	0.2	0	0.672873776	0.266408331
Paleiron, N. (2021)	0.290876242	0.264477129	0.318000682	53.64495035
Pirnay, J. P. (2020)	0.6	0.142048896	0.977994031	0.266408331
Stachow, E. (2021)	0.333333333	0.145171951	0.551405594	1.041414386
Stanila, V. (2020)	0.222222222	0.111184467	0.356746154	2.203923468
Talmy, T. (2021)	0.378151261	0.292864824	0.467353406	5.788326471
Pooled	0.231551907	0.094718873	0.384709896	100
Statistics				
I-squared	93.20497514	88.47318098	95.99435345	
Cochran's Q	88.29989768			
Chi2, p	1.11E-16			

Table S20: Incidence of Headache among Symptomatic COVID-19 Cases

Study	Incidence	LCI 95%	HCI 95%	weight (%)
Kasper, M. R. (2020)	0.6798419	0.64618545	0.71258879	36.78856866
Lalani, T. (2021)	0.8	0.32712622	1	0.266408331
Paleiron, N. (2021)	0.60162602	0.57260664	0.63029292	53.64495035
Pirnay, J. P. (2020)	0.4	0.02200597	0.8579511	0.266408331
Stachow, E. (2021)	0.0952381	0.00208214	0.26630698	1.041414386
Stanila, V. (2020)	0.31111111	0.18311694	0.4550579	2.203923468
Talmy, T. (2021)	0.40336134	0.31667143	0.49315679	5.788326471

Pooled	0.60775221	0.44434412	0.76525779	100
Statistics				
I-squared	92.6041271	87.2860143	95.6977349	
Cochran's Q	81.126326			
Chi2, p	2.11E-15			

Table S21: Incidence of Myalgia among Symptomatic COVID-19 Cases

**Study**	**Incidence**	**LCI 95%**	**HCI 95%**	**weight (%)**
Lalani, T. (2021)	0.4	0.022005969	0.857951104	0.421455939
Paleiron, N. (2021)	0.472448058	0.443083622	0.501908055	84.86590038
Pirnay, J. P. (2020)	0.4	0.022005969	0.857951104	0.421455939
Stachow, E. (2021)	0.095238095	0.002082142	0.266306977	1.647509579
Stanila, V. (2020)	0.133333333	0.047172091	0.25062766	3.486590038
Talmy, T. (2021)	0.235294118	0.163030913	0.316065286	9.157088123
Pooled	0.429527378	0.102031715	0.776905314	100
Statistics				
I-squared	91.69146959	84.68186137	95.49346828	
Cochran's Q	60.17911414			
Chi2, p	1.12E-11			

Table S22: Incidence of Nasal Congestion among Symptomatic COVID-19 Cases

**Study**	**Incidence**	**LCI 95%**	**HCI 95%**	**weight (%)**
Kasper, M. R. (2020)	0.437417655	0.402278782	0.472873038	91.28605769
Lalani, T. (2021)	0.8	0.327126224	1	0.661057692
Stachow, E. (2021)	0.095238095	0.002082142	0.266306977	2.584134615
Stanila, V. (2020)	0.333333333	0.202031572	0.478708911	5.46875
Pooled	0.424228725	0.1466458	0.715897658	100
Statistics				
I-squared	81.48749454	51.82909	92.88548092	
Cochran's Q	16.20526193			
Chi2, p	0.001029225			

Table S23: Incidence of Rhinorrhea among Symptomatic COVID-19 Cases

**Study**	**Incidence**	**LCI 95%**	**HCI 95%**	**weight (%)**
Paleiron, N. (2021)	0.29087624	0.26447713	0.31800068	88.70644774
Stachow, E. (2021)	0.14285714	0.02046135	0.33094585	1.72206648

Talmy, T. (2021)	0.25210084	0.17779688	0.33437228	9.571485783
Pooled	0.28479551	0.23445233	0.3368143	100
Statistics				
I-squared	28.8325083	0	92.5971529	
Cochran's Q	2.81027187			
Chi2, p	0.24533371			

Table S24: Incidence of Sore Throat among Symptomatic COVID-19 Cases

**Study**	**Incidence**	**LCI 95%**	**HCI 95%**	**weight (%)**
Stachow, E. (2021)	0.095238095	0.0020821	0.266307	11.52815
Stanila, V. (2020)	0.222222222	0.1111845	0.3567462	24.396783
Talmy, T. (2021)	0.268907563	0.1927145	0.3525277	64.075067
Pooled	0.238335831	0.1546022	0.3302195	100
Statistics				
I-squared	35.69418876	0	79.344149	
Cochran's Q	3.110138822			
Chi2, p	0.211174736			
